# Ectopic Craniopharyngioma of the Clivus: A Case Report

**DOI:** 10.1159/cro/abzag028

**Published:** 2026-08-22

**Authors:** Qiang Dong, Liang Niu, Jing Shi, Beiyan Tang, Qiang Li, Yawen Pan, Lei Duan

**Affiliations:** a Department of Neurosurgery and Laboratory of Neurosurgery, Lanzhou University Second Hospital, Lanzhou, China; b Key Laboratory of Neurology of Gansu Province, Lanzhou, China; c Department of Intensive Care Unit, Lanzhou University Second Hospital, Lanzhou, China

**Keywords:** Craniopharyngioma, Adamantinomatous craniopharyngioma, Ectopic tumors, Clivus, Case report

## Abstract

**Introduction:**

Craniopharyngioma is a rare benign epithelial tumor that usually arises in the sellar or suprasellar region. Ectopic craniopharyngioma of the clivus is exceptional and may present with nonspecific symptoms that mimic other skull base lesions.

**Case Presentation:**

We report a 56-year-old woman with a 4-year history of intermittent headache and occasional blurred vision. Ophthalmological consultation showed reduced visual acuity, but visual evoked potential testing and fundus photography were unremarkable. Perioperative endocrine testing showed a mildly elevated preoperative thyroid-stimulating hormone level and a postoperative decrease in triiodothyronine, adrenocorticotropic hormone, and cortisol, without documented diabetes insipidus or new neurological deficit. Magnetic resonance imaging and computed tomography demonstrated a cystic lesion in the sellar-clival region with clival thinning and compression. The patient underwent endoscopic endonasal transsphenoidal resection, and histopathology confirmed adamantinomatous craniopharyngioma. Postoperative imaging showed satisfactory resection, and the headache improved.

**Conclusion:**

This case highlights that ectopic craniopharyngioma should be considered in patients with chronic headache and a cystic clival lesion, even when classic sellar symptoms are absent.

## Introduction

Chronic headache is common in clinical practice and is usually primary or benign. However, persistent, progressive, or atypical headache requires careful screening for secondary causes, including infection, vascular disease, inflammatory disease, and intracranial or skull base tumors [1, 2]. Headache accompanied by visual disturbance, anosmia, neck pain, cranial neuropathy, endocrine symptoms, or abnormal imaging should prompt clinicians to broaden the differential diagnosis beyond primary headache disorders [1–3].

Craniopharyngioma is a rare, histologically benign epithelial tumor that arises from remnants of the craniopharyngeal duct or related developmental epithelium [4, 5]. It typically occurs in the sellar or suprasellar region and causes symptoms through compression of the optic apparatus, hypothalamic-pituitary axis, third ventricle, or adjacent neurovascular structures [4–7]. Common manifestations include visual impairment, endocrine dysfunction, hypothalamic symptoms, and increased intracranial pressure [4, 5].

Ectopic craniopharyngioma is uncommon and may arise outside the sellar region along presumed embryologic migration pathways or after prior surgery through implantation or cerebrospinal fluid dissemination [8–11]. Primary clival craniopharyngioma is particularly rare. Its imaging appearance may overlap with chordoma, chondrosarcoma, Rathke cleft cyst, pituitary adenoma, meningioma, metastasis, or inflammatory skull base disease [8, 12]. Accurate preoperative evaluation is therefore important because treatment strategy and prognosis differ across these entities.

We report a rare case of ectopic adamantinomatous craniopharyngioma of the clivus in a patient whose main symptom was long-standing isolated headache with intermittent blurred vision. The objective of this case report is to describe the diagnostic reasoning, differential diagnosis, operative management, pathological findings, and clinical implications of this unusual presentation. The CARE Checklist has been completed by the authors for this case report, attached as online supplementary material (for all online suppl. material, see https://doi.org/10.1159/cro/abzag028).

## Case Presentation

A 56-year-old woman reported intermittent headache without an obvious precipitating factor for 4 years. The headache was occasionally accompanied by blurred vision. She denied dizziness, nausea, vomiting, limb weakness, sensory disturbance, or cerebellar symptoms. The symptom was not initially considered severe. Six months before admission, the headache worsened without an obvious trigger, and she was admitted to our hospital for further evaluation.

Her medical history included hypertension for more than 7 years. She regularly took amlodipine besylate, rosuvastatin, and aspirin. Neurological examination did not reveal deficits in other cranial nerves, motor function, sensation, or cerebellar function. Ophthalmological consultation showed visual acuity of 0.3 in the right eye and 0.6 in the left eye. Intraocular pressure was 10.6 mm Hg in the right eye and 12.5 mm Hg in the left eye. The corneas were clear, the anterior chambers were moderately deep and clear, the pupils were equal and round with a diameter of approximately 3 mm, and light reflexes were present. Mild lens opacity was noted. Visual field testing was unreliable because of poor cooperation, while visual evoked potential testing and fundus photography showed no abnormality. The ophthalmology service recommended treatment of the primary disease and outpatient ophthalmological follow-up.

Preoperative endocrine testing showed a mildly elevated thyroid-stimulating hormone level, while the remaining recorded pituitary-related endocrine values were within the laboratory reference ranges or compatible with menopausal status. The perioperative endocrine laboratory findings are summarized in Table 1.

**Table 1.** Perioperative endocrine laboratory findings

Hormone	Abbrev.	Preoperative	Postoperative	Unit	Reference range	Interpretation
Triiodothyronine	T3	1.44	0.98 ↓	nmol/L	1.02–2.96	Postoperative decrease below the reference range
Thyroxine	T4	118.30	95.40	nmol/L	55.47–161.25	Within reference range
Thyroid-stimulating hormone	TSH	5.572 ↑	1.576	μIU/mL	0.38–4.34	Mildly elevated preoperatively; normalized postoperatively
Prolactin	PRL	9.59	6.84	ng/mL	2.80–29.20	Within reference range
Follicle-stimulating hormone	FSH	84.03	53.10	mIU/mL	Menopause range varies by laboratory	Compatible with menopausal status in this 56-year-old patient
Luteinizing hormone	LH	27.32	20.22	mIU/mL	Menopause range varies by laboratory	Compatible with menopausal status in this 56-year-old patient
Adrenocorticotropic hormone	ACTH	24.70	<5.00 ↓	pg/mL	5.00–46.00 (morning); 5.00–23.00 (afternoon)	Postoperative decrease; requires clinical correlation
Growth hormone	GH	0.27	1.43	ng/mL	0.00–5.00	Within reference range
Insulin-like growth factor-1	IGF-1	118.00	105.00	ng/mL	31.00–323.00	Within reference range
Cortisol	COR	11.20	3.77	μg/dL	5.00–25.00 (morning); 2.50–12.50 (afternoon)	Lower postoperative value; interpretation depends on sampling time

Arrows indicate values outside the listed laboratory reference range. Cortisol interpretation depends on sampling time.

The clinical reasoning before magnetic resonance imaging (MRI) was based on the chronic progressive course, the patient’s age, the recent aggravation of headache, and intermittent blurred vision. These features represented red flags for a secondary headache disorder rather than a purely primary headache syndrome. Skull base or sellar-clival disease was therefore considered, and MRI was obtained to evaluate possible intracranial and skull base pathology. MRI showed a cystic lesion in the sellar-clival region. The lesion showed short T1 and slightly long T2 signal, slightly low signal on diffusion-weighted imaging, and no obvious enhancement. The lesion measured approximately 2.1 × 2.4 × 2.1 cm. Computed tomography showed thinning of the clival bone with local compression and absorption. Postoperative MRI showed satisfactory resection without obvious residual lesion (Fig. 1).

**Fig. 1.**
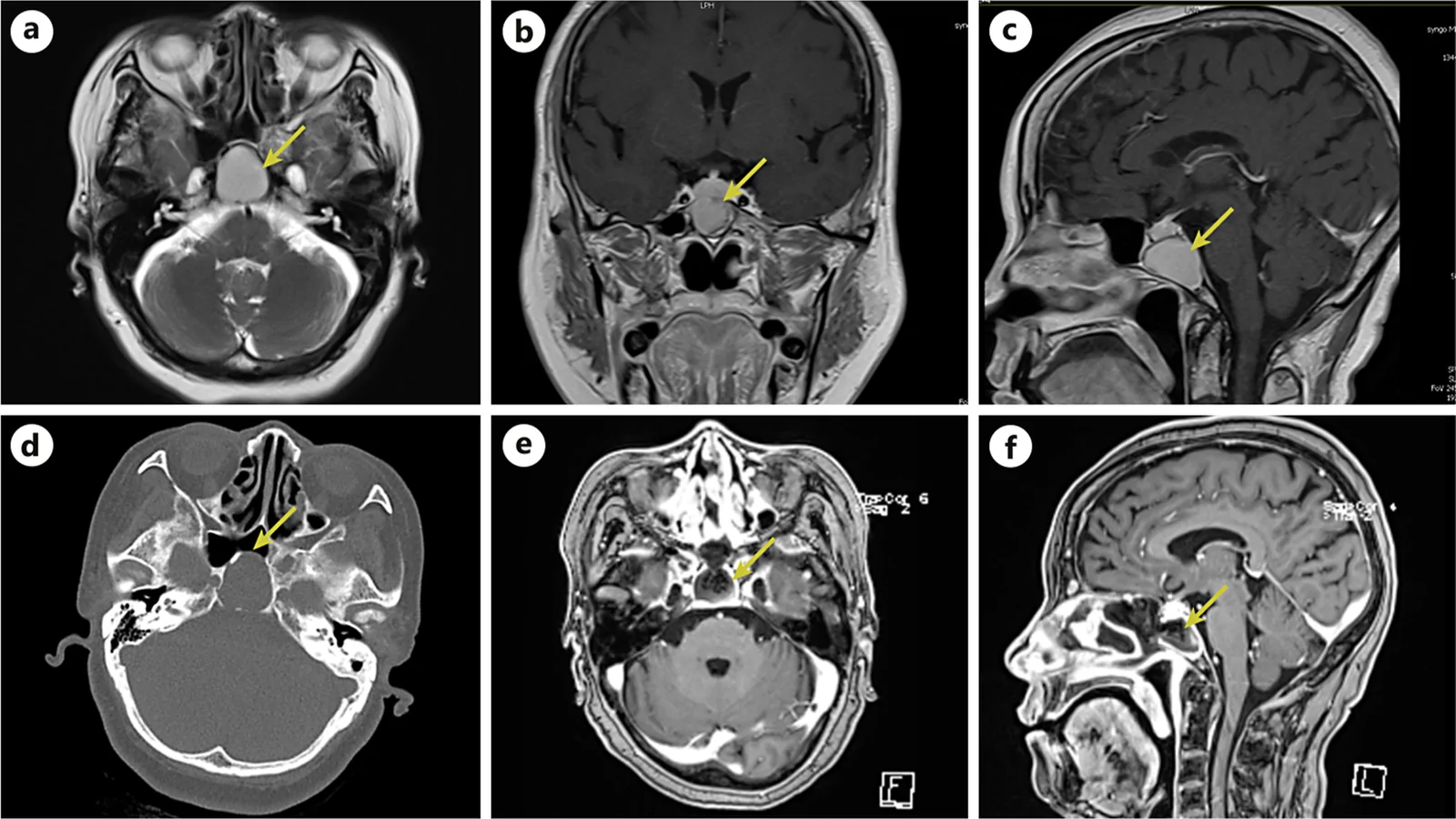
Preoperative and postoperative imaging of the clival lesion. **a–c** Preoperative magnetic resonance images show a cystic lesion in the sellar-clival region, with short T1 and slightly long T2 signal and no obvious enhancement. **d** Preoperative computed tomography shows thinning of the clivus with local compression and absorption. **e**, **f** Postoperative magnetic resonance images show satisfactory resection without obvious residual lesion. Yellow arrows indicate the lesion or postoperative tumor bed.

### Differential Diagnosis

The main preoperative differential diagnosis included chordoma and chondrosarcoma because these are among the most common primary clival tumors. Chordoma often arises from notochordal remnants and may produce destructive midline clival lesions, whereas chondrosarcoma more often has an off-midline petroclival origin. Both can present with headache and cranial neuropathy, and both require different long-term management from craniopharyngioma.

Sellar and parasellar cystic lesions were also considered. Rathke cleft cyst and cystic pituitary adenoma may show cystic sellar or suprasellar components and can cause headache or visual symptoms. Meningioma, metastasis, inflammatory lesions, and mucoceles were considered because they can involve the skull base or sphenoid-clival region. Ectopic craniopharyngioma was included because the lesion was cystic, located along a plausible developmental pathway related to Rathke pouch remnants, and showed imaging characteristics compatible with a proteinaceous or cholesterol-rich cystic tumor [8, 12]. Because imaging was not sufficiently specific and the patient had persistent symptoms, surgery was selected to obtain a histological diagnosis, decompress the lesion, and prevent further neurological deterioration.

### Treatment and Outcome

Endoscopic endonasal transsphenoidal resection of the clival tumor was performed under general anesthesia. A nasal mucosal flap was prepared, the sphenoid sinus opening was identified, and the anterior wall of the sphenoid sinus was drilled. A hemispherical cystic tumor protruded into the sphenoid sinus cavity at the lower clivus. The cyst wall was grayish-red and tough.

After the anterior wall of the tumor was opened under endoscopic visualization, grayish-brown granular fluid drained from the lesion. The tumor was resected endoscopically while adjacent neurovascular structures were protected. The sellar floor was reconstructed. Postoperative management included anti-infective treatment, nutritional support, and symptomatic treatment. Histopathological examination confirmed adamantinomatous craniopharyngioma (Fig. 2).

**Fig. 2.**
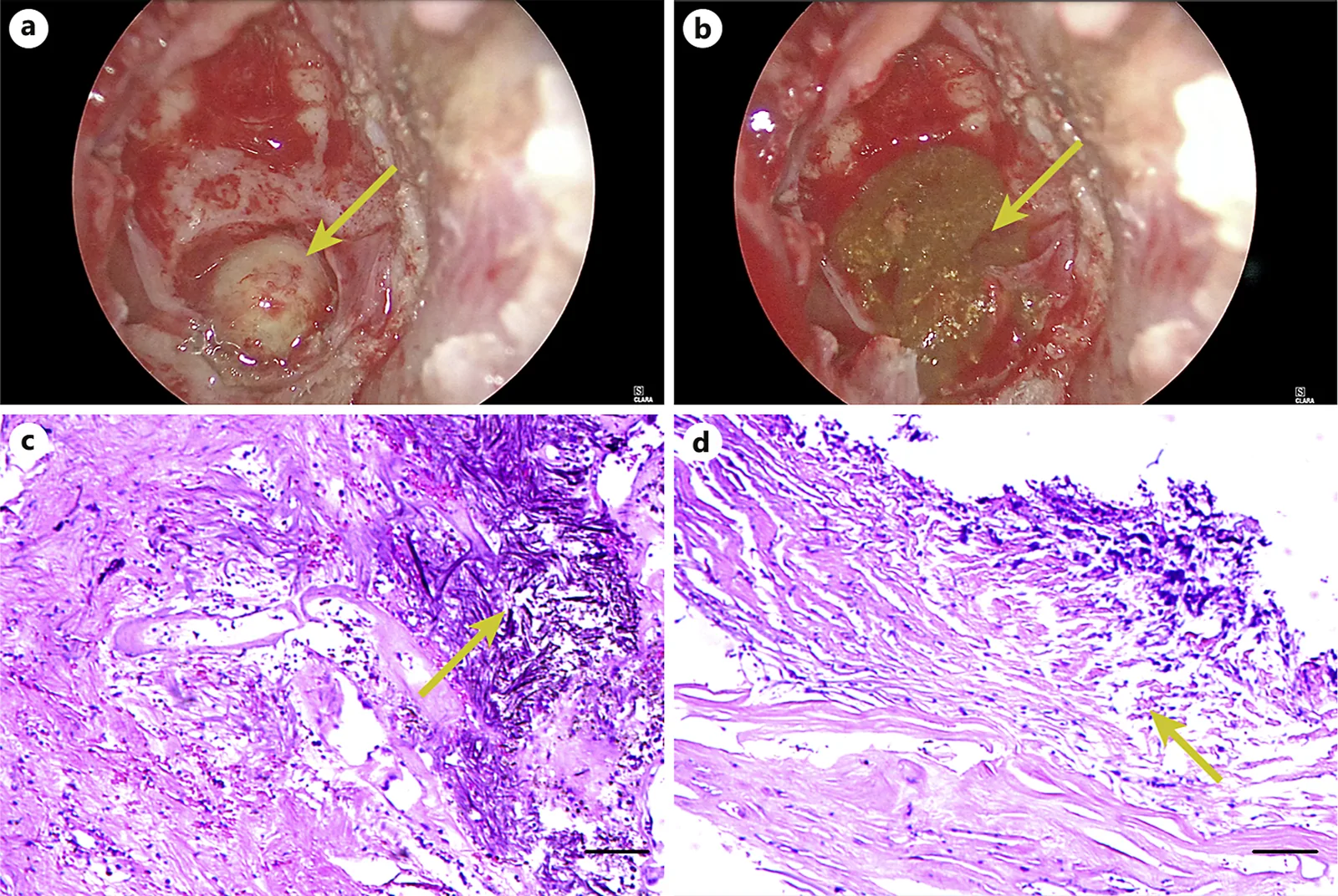
Endoscopic intraoperative and histopathological findings. **a**, **b** Intraoperative endoscopic images show a hemispherical cystic tumor at the clivus. The cyst wall was grayish-red and tough, and grayish-brown granular fluid was observed after opening the anterior wall. **c**, **d** Hematoxylin and eosin staining shows a fibrous cyst wall with hyaline degeneration, cholesterol crystals, and inflammatory cell infiltration. Yellow arrows indicate the lesion cavity and representative pathological features.

The postoperative course was uneventful. The patient’s headache was significantly relieved compared with the preoperative condition. Postoperative cranial imaging showed satisfactory tumor resection without obvious residual lesion. Postoperative endocrine testing showed decreases in T3, adrenocorticotropic hormone, and cortisol compared with the corresponding preoperative values (Table 1). These findings require clinical correlation and follow-up. No postoperative diabetes insipidus, electrolyte disturbance, or new neurological deficit was documented. She was discharged in stable general condition. No standardized headache score or quality-of-life scale had been obtained before surgery, so postoperative improvement was assessed clinically.

## Discussion

This case illustrates a rare ectopic clival adamantinomatous craniopharyngioma presenting mainly as long-standing headache. The presentation differed from the typical clinical spectrum of sellar and suprasellar craniopharyngiomas, which commonly includes visual impairment, endocrine dysfunction, hypothalamic symptoms, or hydrocephalus-related intracranial pressure [4–7]. Although the patient reported intermittent blurred vision and had reduced visual acuity on ophthalmological consultation, visual evoked potential testing and fundus photography were normal, and visual field testing was unreliable. Preoperative endocrine testing showed only mildly elevated TSH among the recorded pituitary-related values. These findings help explain why the lesion was not clinically recognized as a classic sellar-region craniopharyngioma before imaging.

The clival location is the central diagnostic feature of this case. Ectopic craniopharyngiomas have been described in the clivus, fourth ventricle, optic pathway, posterior fossa, brain parenchyma, spine, and postoperative implantation sites [8–17]. Primary ectopic tumors may reflect misplaced embryonic epithelium or tumorigenesis outside the sella. Secondary ectopic lesions can occur through surgical tract implantation, cerebrospinal fluid dissemination, or meningeal spread [9–11]. Our patient had no history of previous craniopharyngioma surgery, supporting a primary ectopic lesion.

The clinical workup required distinction from common clival and sellar-clival lesions. Chordoma and chondrosarcoma are important alternatives because they are more typical clival tumors and may require different surgical and adjuvant strategies. Rathke cleft cyst, cystic pituitary adenoma, meningioma, metastasis, and inflammatory lesions should also be considered when evaluating a cystic sellar-clival lesion. In this case, histopathology was decisive and confirmed adamantinomatous craniopharyngioma.

The pathology also helps explain the unusual symptom course. Adamantinomatous craniopharyngioma is characterized by cystic components, cholesterol-rich contents, calcification, inflammatory reaction, and epithelial elements [4, 18, 19]. Long-standing quiescence followed by symptom aggravation may reflect cyst fluid changes, intermittent inflammation, microhemorrhage, or mild volume increase reaching a threshold for dural or skull base irritation. These mechanisms remain inferential in this single case, but they provide a plausible explanation for chronic headache without severe visual pathway findings or overt preoperative hypopituitarism.

The main clinical lesson is that persistent headache with visual complaints and a clival cystic lesion should not be attributed only to primary headache or nonspecific skull base disease. Ectopic craniopharyngioma should be included in the differential diagnosis, especially when imaging suggests a proteinaceous or cholesterol-rich cystic lesion near the sellar-clival region. Endoscopic endonasal surgery can provide both tissue diagnosis and lesion removal in selected cases, but management should be individualized according to tumor extent, neurovascular relationships, endocrine and visual status, and patient comorbidities.

This report has limitations. Formal quantitative headache and quality-of-life scales were not available, and postoperative improvement was therefore described clinically rather than by standardized scores. Visual field testing was unreliable because of poor cooperation, although visual evoked potential testing and fundus photography were unremarkable. Endocrine testing was available before and after surgery, but longer serial endocrine follow-up is needed to determine whether the postoperative decreases in T3, adrenocorticotropic hormone, and cortisol are transient perioperative changes or clinically meaningful pituitary-adrenal axis abnormalities. Longer imaging follow-up is also needed because craniopharyngioma can recur, and ectopic lesions may pose diagnostic and surveillance challenges.

## Conclusion

Ectopic adamantinomatous craniopharyngioma of the clivus is rare and may present with chronic headache rather than classic sellar symptoms. Careful attention to red flags, sellar-clival imaging, and a broad differential diagnosis is essential. Histopathological confirmation remains critical, and endoscopic endonasal surgery may be an effective diagnostic and therapeutic option in selected patients.

## Statement of Ethics

This study was conducted according to the guidelines of the Declaration of Helsinki and was approved by the Ethics Committee of Lanzhou University Second Hospital (Approval No. 2025A-302). Written informed consent was provided by the patient for use of clinical data and information and for publication.

## Conflict of Interest Statement

The authors have no conflicts of interest to declare.

## Funding Sources

This work was supported by grants from the National Natural Science Foundation of China (82560469), the Natural Science Foundation of Gansu Province (24JRRA1088/26JRRA797), the Project of the Health and Family Planning Commission of Gansu (GSWSKY2024-41), Cuiying Scientific and Technological Innovation Program of the Second Hospital and Clinical Medical SchoolLanzhou University (CY2025-HL-A05), and the Key Incubation Project Funds of the Second Hospital and Clinical Medical School, Lanzhou University (2025-25-zdfy-020).

## Author Contributions

Qiang Dong and Liang Niu contributed to conceptualization. Yawen Pan and Lei Duan acquired funding. Jing Shi and Qiang Li contributed to investigation. Beiyan Tang and Liang Niu contributed to supervision and writing – review and editing. Qiang Dong and Lei Duan wrote the original draft. All authors reviewed and approved the final manuscript.

## Data Availability

All data generated or analyzed during this study are included in this article. Further inquiries can be directed to the corresponding author.
